# The centrality of lived space in advancing SDG 3 through Community-Led Monitoring (CLM) in Asia-Pacific

**DOI:** 10.3389/fpubh.2025.1670863

**Published:** 2025-11-10

**Authors:** Maya Dania, Harry Prabowo, Reni Juwitasari, Matthew Kusen

**Affiliations:** 1 School of Social Innovation, Mae Fah Luang University, Chiang Rai, Thailand; 2 APN+/Seven Alliance, Bangkok, Thailand; 3 ARCID, School of Social Innovation, Mae Fah Luang University, Chiang Rai, Thailand; 4 Health Equity Matters, Sydney, NSW, Australia

**Keywords:** Community-Led Monitoring (CLM), SDG 3, Asia-Pacific, communicable disease, health equity

## Introduction

Sustainable Development Goal 3 (SDG 3) aims to achieve universal health and wellbeing, including the elimination of AIDS, tuberculosis, malaria, and other communicable diseases by 2030 (1). Rooted in a global ethic that all people are entitled to health (2, 3), it envisions shared global responsibility. However, in the Asia-Pacific region, health governance remains largely technocratic and state-focused, often overlooking the lived experiences of marginalized populations.

SDG 3 thus reflects an abstract form of cosmopolitanism (4): a universal view detached from the political and spatial factors influencing health outcomes. Community-Led Monitoring (CLM) offers a grounded correction, translating global commitments into locally actionable practices. Based on Lefebvre's theory of lived space (5) and critical cosmopolitan thought (6–10), CLM is a community-driven process that generates evidence, reveals exclusion, and boosts accountability.

In this article, we (1) connect cosmopolitan and spatial theory to public health outcomes, (2) synthesize evidence from Asia-Pacific CLM experiences, and (3) outline how CLM can be institutionalized and sustainably funded within health systems. We argue that CLM embodies grounded cosmopolitanism, amplifying marginalized voices and transforming the universal aims of SDG 3 into equitable, everyday practices.

## Methods and limitations

This article utilizes secondary evidence from the Asia-Pacific CLM Resource Repository (11), which was created by the Community of Practice on Community-Led Monitoring in collaboration with UNAIDS, the Seven Alliance, and the CDC. The repository compiles case studies, technical briefs, and policy reports from over 15 countries. Cases were selected based on three criteria: (a) documented CLM implementation in HIV, TB, or malaria programs; (b) engagement with national or subnational health systems; and (c) evidence of outcomes for key populations. The chosen examples represent regional diversity and various political contexts. The qualitative analysis highlights recurring themes, including stigma in clinical settings, insufficient service integration, and barriers to digital access, as well as notable differences across different contexts.

Limitations include variations in data quality caused by differences in community capacity, donor influence, and political constraints. In restrictive environments like Myanmar or Pakistan, surveillance risks and limited access to formal health systems hinder monitoring. Donor dependence raises concerns about sustainability, and community participation risks tokenism when power-sharing remains superficial and unbalanced. As a secondary review, the study cannot independently verify data but interprets reported experiences to derive regional lessons.

Despite these limitations, CLM remains a vital accountability tool that provides experiential evidence, strengthens community agency, and uncovers systemic inequities in health governance.

## Regional evidence and community-led accountability in health systems

Building on the theoretical framework of cosmopolitanism and lived space, this section presents regional evidence showing how Community-Led Monitoring (CLM) functions as a practical form of accountability within Asia-Pacific health systems. It examines how communities transform lived experiences into evidence, challenge institutional exclusion, and influence policy processes that support Sustainable Development Goal 3 (SDG 3).

Kant's Perpetual Peace considered universal dignity the foundation of cosmopolitan rights (2). However, depending on states to realize these ideals often makes them seem abstract. Later thinkers paid closer attention to inequality in cosmopolitanism: Habermas emphasized transnational deliberation (9); Benhabib described “democratic iterations” that localize universal norms (6); Appiah proposed “rooted cosmopolitanism” that balances global ethics with local culture (7); Cheah viewed cosmopolitanism as subaltern praxis built through struggle (8); and Fraser argued that justice requires redistribution, recognition, and representation (10).

For public health, the lesson is clear: universal goals like SDG 3 cannot rely solely on technical indicators but must include mechanisms that address inequality within everyday health systems. Community-Led Monitoring (CLM) achieves this by collecting evidence from those most impacted, revealing service gaps, combating stigma, and turning global ideals into tangible progress toward health equity.

Regional data from the Asia-Pacific CLM Repository (11) highlight ongoing challenges, including financial instability, political opposition, stigma, and infrastructural gaps. Without community-led accountability, the goal of SDG 3 remains at risk. In Cambodia, Indonesia, Myanmar, and Papua New Guinea, CLM monitors report systemic issues such as denied HIV services, lack of viral-load testing, discriminatory practices, and geographic barriers, revealing deep-rooted structural inequalities. Conversely, supportive environments like Thailand and Nepal have integrated CLM into their national TB and HIV strategies, while repression in Myanmar and Pakistan has hindered participation.

The COVID-19 pandemic worsened these vulnerabilities: diagnostic resources were diverted, staff were redeployed, and community monitoring was halted, especially in conflict-affected states (12, 13). These disruptions revealed both the fragility of technocratic health systems and the limitations of universal health goals without community participation.

Through Cheah's concept of praxis (8), CLM demonstrates cosmopolitanism from below, emerging from communities facing exclusion and transforming lived experiences into evidence and accountability. It shifts epistemic authority and guarantees representation where it is often missing. In Fraser's terms (10), such practice achieves justice by connecting redistribution, recognition, and representation.

CLM redefines how SDG 3 is implemented by turning abstract commitments into collaboratively developed systems of care that support marginalized groups. It tackles the structural conflict between transnational ideals and national implementation by incorporating community evidence into governance. Unlike frameworks such as the WHO or Global Fund, CLM formalizes community-generated data as credible evidence for decision-making. However, its success relies on sustainability and safeguarding against co-option or donor dependence. Recognizing both strengths and risks is crucial if CLM is to shift from a corrective ideal to a sustainable practice for health equity (14).

Understanding both the strengths and vulnerabilities of CLM is crucial for its role in promoting fair health governance. When institutionalized and supported by robust legal and financial systems, CLM translates global commitments into practical care systems that benefit marginalized populations. Without these supports, it risks remaining only symbolic or disconnected. To expand this analysis, the following section uses Lefebvre's spatial framework to explore how CLM reshapes the lived spaces of health across the Asia-Pacific, changing the everyday geographies where exclusion, accountability, and justice are discussed.

## Spatial dimensions of community-led monitoring in Asia-Pacific health governance

Building on the regional evidence presented earlier, this section adopts a spatial perspective to explain how Community-Led Monitoring (CLM) reshapes the environments in which health rights are claimed and challenged. Drawing on Henri Lefebvre's concept of socially produced space (5), this view regards health governance not merely as a neutral system of services and data, but as a dynamic social space shaped by power, policy, and everyday struggles. With this perspective, CLM works within the lived spaces of communities, where stigma, inequality, and exclusion are most apparent. Recognizing these spatial aspects helps explain how CLM turns technical monitoring into a political act of accountability, connecting global health goals with local realities in the pursuit of SDG 3.

Henri Lefebvre's spatial theory argues that space is not neutral but is socially constructed through historical and political struggles (5). His spatial triad, which includes spatial practice (perceived space), representations of space (conceived space), and representational spaces (lived space), illustrates how each component influences the organization and experience of space. When these parts are considered separately, space loses its political importance (15).

This framework sheds light on health governance. Conceived space reflects the rationalities of planners and institutions (16), where SDG 3 is implemented through global indicators and national dashboards (17). While these abstractions establish shared goals, they often disconnect from real lived experiences. Lived space, in contrast, reveals how communities face inequality, exclusion, and struggle.

Community-Led Monitoring (CLM) depends on the lived experiences of its participants. By recording stigma, service gaps, and access issues, CLM transforms personal stories into evidence that challenges technocratic control and reintroduces politics into health governance. Without this foundation, SDG 3 risks continuing the inequalities it aims to eliminate.

Across the Asia-Pacific, CLM is a spatial practice rooted in daily struggles for recognition and rights. Regional evidence shows three common patterns:

1. Persistent stigma and discrimination, from Cambodia to India, violate privacy and deny services to people living with HIV and TB (18, 19).
2. Weak institutional recognition in Cambodia, Nepal, and Sri Lanka hampers accountability and policy adoption (18, 20, 21).
3. Structural barriers, including digital inequality in Indonesia, PNG, and Mongolia, restrict monitoring reach (22–24).

Different contexts affect outcomes. Thailand has partially integrated CLM into universal health coverage, while Myanmar and Pakistan face repression and legal barriers (25, 26). In the Philippines and Vietnam, fragmented programs and the criminalization of key populations hinder data integration (27–29).

When governments prioritize community evidence, CLM improves accountability and inclusiveness; when voices are ignored, SDG 3 remains vague. This distinction differentiates CLM from the WHO and Global Fund frameworks, which rely on standardized reporting. By institutionalizing community data, CLM shifts accountability downward but still faces risks of donor dependence, tokenism, and state co-option.

Country experiences highlight these tensions. Along Thailand's northern border, networks such as RSAT and Mplus face challenges, including malaria resurgence and service interruptions, despite their inclusion in policies (30). In PNG, stigma, digital gaps, and volunteer attrition weaken CLM networks like Igat Hope (23). Sri Lanka's community groups lack national recognition (21), while punitive laws in Pakistan restrict organizations such as Nai Zindagi (26). In Mongolia, stigma prevents TB survivors from participating (24); in Vietnam, criminalization of sex work and drug use limits policy discussions (29). Lao PDR's silence on key populations (31), Bangladesh's legal ambiguity (32, 33), and Malaysia's restrictive laws (34) all hinder data collection and advocacy efforts. In Pacific Island nations like Fiji, isolation and chronic underfunding worsen these barriers (35).

Collectively, the experiences of these countries demonstrate that CLM alters health governance by acknowledging lived space as a legitimate source of knowledge and action. When governments accept community-generated evidence, accountability improves, and health systems become more inclusive and responsive to the needs of their communities. On the other hand, when participation encounters legal, political, or digital obstacles, SDG 3 remains vague and unevenly realized.

Understanding these spatial dynamics shows that CLM is more than just a monitoring tool. Significantly, it also changes how health systems value community knowledge and shift decision-making power. This spatial perspective sets the stage for the subsequent discussion, which explores how these insights can be used in regional policies and strategies to incorporate CLM into broader public health governance and equity frameworks.

## Discussion

Achieving Sustainable Development Goal 3 (SDG 3) requires moving beyond technocratic planning and adopting practices rooted in lived realities. Although SDG3′s vision of health equity, particularly the elimination of HIV, tuberculosis, and malaria, is morally compelling, its implementation remains focused on national indicators and clinical benchmarks (36). These metrics fail to account for lived space (5), which we define here as the embodied contexts where key populations receive or are denied care. The Asia-Pacific experience with Community-Led Monitoring (CLM) demonstrates that lived space is both a site of exclusion and a potential space for change (37).

Unlike the accountability models of the WHO or Global Fund, which depend on standardized metrics, CLM incorporates accountability through community experience. It turns experiential evidence into decision-making power, shifting epistemic authority and transforming how health systems engage with marginalized groups.

Table 1 summarizes CLM initiatives across 13 countries, highlighting community-driven tools, effects on key populations, and the conditions that shift CLM from symbolic participation to transformative accountability.

**Table 1:** Comparative overview of Community-Led Monitoring initiatives in the Asia-Pacific.

**Country**	**CLM centrality**	**Impact on key populations**
Cambodia (18)	Digital feedback system for stigma, TB drug side effects, and access gaps	Policy changes on social protection, inclusion, and reduced stigma
Philippines (28)	CareTB App, CallKaLungs hotline, TB Scorecard for community advocacy	Influenced local health policy, reduced discrimination
Indonesia (38)	Community-driven health monitoring via CLM-INA Task Force	Strengthened local partnerships, extended CLM reach
India (39)	Capacity-building, multi-stakeholder co-creation workshops	Empowered communities, built second-line leadership
Myanmar (25)	Trust-building among community networks for healthcare monitoring	Enhanced collective implementation through networks
Nepal (20)	Rights-based service monitoring by marginalized Rights Holders	Policy alignment with SDGs attracted government support
Thailand (40)	TB-specific CLM with integration into the National TB Program	Improved strategic planning and sustainability
Papua New Guinea (PNG) (41)	CLM data integrated into the national HIV M&E system	Increased credibility, enabled government engagement
Sri Lanka (42)	Pilot-informed CLM models tailored to key populations	Effective participation, long-term adaptable models
Bhutan (43)	Toolkit adaptation balancing tech use and community engagement	Inclusive monitoring with open-source data systems
Pakistan (32)	Peer monitoring of HIV service delays and stigma	Improved grievance mechanisms, staff behavior
Bangladesh (33)	CLM for people who use drugs and street-based key populations	Improved outreach and minimized service dropouts
Malaysia (34)	Piloting CLM to bridge gaps between health workers and transgender people	Improved service alignment with the trans community's needs

Across Cambodia, the Philippines, and Indonesia, CLM relies on digital feedback systems and advocacy platforms to document stigma, side effects, and service gaps (18, 28, 38). In India and Nepal, it emphasizes capacity-building and rights-based monitoring that empower marginalized groups (20, 39). Thailand and Papua New Guinea demonstrate advanced integration, incorporating CLM into national TB and HIV frameworks to enhance sustainability (40, 41). These examples demonstrate that CLM serves as both a grassroots accountability tool and, when institutionalized, as part of the national health system.

Where CLM mechanisms garner recognition, they create tangible impacts, as demonstrated by social protection reforms in Cambodia, grievance redress in Pakistan, decreased discrimination in the Philippines, and enhanced service coordination for transgender communities in Malaysia (18, 28, 32, 34). These results distinguish CLM from participatory monitoring and community health worker models by transforming community evidence into enforceable policy influence.

However, differences still exist. In Myanmar, repression hampers monitoring (25); in Bhutan and Sri Lanka, CLM remains in pilot stages (42, 43); and in Bangladesh, legal uncertainties restrict outreach to people who use drugs (33). Without supportive political and legal frameworks, CLM may remain fragmented. Three regional lessons emerge:

1. Institutionalizing within national M&E frameworks, as seen in Thailand and PNG, enhances credibility and long-term sustainability.
2. Investing in leadership for marginalized populations, as demonstrated in India and Nepal, enhances ownership.
3. Legal recognition of community data, seen in Cambodia, the Philippines, and Malaysia, allows for large-scale policy influence.

CLM centers treat lived experience as valid evidence. It shifts accountability from mere compliance to active participation, thereby linking universal health goals to everyday realities. When incorporated into governance, CLM not only enhances accountability but also transforms the environments where health rights are debated. In Lefebvrian terms, it alters social space by documenting exclusion while reshaping the landscape of health equity.

Meanwhile, CLM highlights the limitations of SDG 3 as an abstract, global initiative that may overlook local needs. In contrast, CLM practices rooted in cosmopolitanism promote local agency, cross-scalar solidarity, and accountability based on lived experience. Networks of peer educators, civil society coalitions, and national partnerships demonstrate that CLM can move beyond critique to become a recognized, data-driven approach. Therefore, three strategies are crucial to driving this transformation.

1. Integrate CLM into national health systems to maintain continuity and establish credibility.
2. Secure long-term funding through government budgets, reducing dependence on donors.
3. Protect civic space to foster genuine participation.

Despite advances, challenges still exist. Short-term funding, political backlash, and data gaps threaten credibility. Preventing tokenistic participation needs long-term efforts, legal safeguards, and systems that ensure community evidence influences meaningful policy decisions.

In conclusion, CLM is not just supplementary but transformative, as it links universal rights to local care practices. Embedding it within health governance can make the moral commitment of SDG 3 tangible, inclusive, and enduring.
